# Bed Rest versus Early Ambulation with Standard Anticoagulation in The Management of Deep Vein Thrombosis: A Meta-Analysis

**DOI:** 10.1371/journal.pone.0121388

**Published:** 2015-04-10

**Authors:** Zhenlei Liu, Xixi Tao, Yuexin Chen, Zhongjie Fan, Yongjun Li

**Affiliations:** 1 Department of Surgery, Peking Union Medical College Hospital, Peking Union Medical College & Chinese Academy of Medical Science, Beijing, China; 2 Department of Medicine, Peking Union Medical College Hospital, Peking Union Medical College & Chinese Academy of Medical Science, Beijing, China; 3 Department of Vascular Surgery, Peking Union Medical College Hospital, Peking Union Medical College & Chinese Academy of Medical Science, Beijing, China; 4 Department of Cardiology, Peking Union Medical College Hospital, Peking Union Medical College & Chinese Academy of Medical Science, Beijing, China; University of Messina, ITALY

## Abstract

**Introduction:**

Bed rest has been considered as the cornerstone of management of deep vein thrombosis (DVT) for a long time, though it is not evidence-base, and there is growing evidence favoring early ambulation.

**Methods:**

Electronic databases including Medline, PubMed, Cochrane Library and three Chinese databases were searched with key words of “deep vein thrombosis”, “pulmonary embolism”, “venous thrombosis”, “bed rest”, “immobilization”, “mobilization” and “ambulation”. We considered randomized controlled trials, prospective or retrospective cohort studies that compared the outcomes of acute DVT patients managed with early ambulation versus bed rest, in addition to standard anticoagulation. Meta-analysis pertaining to the incidence of new pulmonary embolism (PE), progression of DVT, and DVT related deaths were conducted, as well as the extent of remission of pain and edema.

**Results:**

13 studies were included with a total of 3269 patients. Compared to bed rest, early ambulation was not associated with a higher incidence of new PE, progression of DVT, or DVT related deaths (RD −0.03, 95% CI −0.05∼ −0.02; Z = 1.24, *p* = 0.22; random effect model, Tau^2^ = 0.01). Moreover, if the patients suffered moderate or severe pain initially, early ambulation was related to a better outcome, with respect to remission of acute pain in the affected limb (SMD 0.42, 95%CI 0.09∼0.74; Z = 2.52, *p* = 0.01; random effect model, Tau^2^ = 0.04). Meta-analysis of alleviation of edema cannot elicit a solid conclusion because of significant heterogeneity among the few studies.

**Conclusions:**

Compared to bed rest, early ambulation of acute DVT patients with anticoagulation was not associated with a higher incidence of new PE, progression of DVT, and DVT related deaths. Furthermore, for the patients suffered moderate or severe pain initially, a better outcome can be seen in early ambulation group, regarding to the remission of acute pain in the affected limb.

## Introduction

DVT is a common disease, with the incidence being up to 0.5/1000 person-years [1]. Due to its relatively high incidence and possible fatal complications, the treatment of DVT has long been a major concern for many physicians and surgeons. The first treatment of DVT can be dated back to 13^th^ century, and both medical and surgical therapies had been studied and adopted in the following centuries [2].

Strict bed rest had been considered as the cornerstone of treatment of acute DVT for a long period, not only because of the concern of possible dislodging thrombosis, but also the confinement to bed with 24 hours unfractionated heparin infusion. However, this treatment was not evidence-based. More and more doctors began to recognize the risk of blood stasis associated with bed rest. Thanks to the introduction of low molecular weight heparin (LMWH) in 1990s, DVT patients can be treated as outpatients [2]. The tradition of immobilization therapy is being challenged by the growing evidence favoring early ambulation.

Several randomized controlled trials (RCTs) and prospective registries had investigated the outcomes of DVT patients treated with early ambulation or bed rest, including PE, progression of DVT, improvement of pain and edema [3, 4, 5]. Although the guideline of DVT treatment in China (2^nd^ edition) [6] did not mention early ambulation for the patients, early ambulation under feasible circumstance was suggested in the 9^th^ guideline of antithrombotic therapy by American College of Chest Physicians (ACCP) [7].

On the basis of the published studies so far, we performed this systematic meta-analysis to further demonstrate the influence of early ambulation versus bed rest on patients with acute DVT.

## Materials and Methods

### Eligibility criteria

(1) RCTs, prospective or retrospective cohort studies with good methodological design. (2) All participants were in the acute phase of DVT at recruitment. (3) Interventions were “bed rest” versus “early ambulation”, in addition to standard anticoagulation. (4) Endpoints were new PE (symptomatic or asymptomatic PE confirmed with CT scan or scintigraphy), progression of DVT (assessed by ultrasound or phlebography) or other DVT related parameters (e.g., extent of pain and edema).

### Literature search

Literatures published up to November 2014 were searched in the following databases: Embase, Medline, PubMed, Cochrane Library, Sinomed, WanFangData and Chinese National Knowledge Infrastructure (CNKI). The last three were used to search for Chinese literatures. Key words included “deep vein thrombosis”, “pulmonary embolism”, “venous thrombosis”, “bed rest”, “immobilization”, “mobilization” and “ambulation”. The synonyms in Chinese were searched in Chinese databases. In addition, references of relative articles were also examined to make sure all the articles relative to our analysis were retrieved.

### Study selection

Title and abstract review was first conducted to rule out the articles apparently mismatched to our eligibility criteria. Then the articles would be examined thoroughly to determine whether or not they should be included for the meta-analysis according to the eligibility criteria. Reviews, former meta-analyses and opinions about the disease were also kept for useful information. All the screening work was conducted independently by two authors, Liu and Tao. Disagreements were discussed and consulted until a consensus was made.

### Statistical analysis

This meta-analysis was conducted with the software, Review Manager (RevMan) version 5.3, which is for Cochrane reviews. Publication bias was tested with visual inspection of funnel plot (in RevMan version 5.3), as well as Begg’s and Egger’s test (in Stata version 12.1). Quality assessment was conducted with risk of bias table for RCT in RevMan, and Newcastle-Ottawa Scale for cohort studies. Heterogeneity of included studies was tested with Chi^2^ and heterogeneity index, I^2^.

We performed the meta-analysis about endpoints consisting of new PE, progression of DVT and deaths, which were dichotomous, using the fixed effect model with Mantel-Haenszel method. Subgroup and sensitivity analyses were also conducted for heterogeneity exploration. Random effect model was used if a solid conclusion cannot be drawn. Risk Difference (RD) (in some studies, Risk Ratio was not applicable because no end events happened), 95% confidence interval (CI) and *p* value were calculated.

The meta-analysis about relief of pain (measured by change of visual analogue scales (VAS), in which patients specify their level of pain by indicating a position along a continuous line between two endpoints. General definition (10 as the highest score): mild pain, VAS<3; moderate pain, VAS 4–6; severe pain, VAS 7–10.) and edema (measured by change of circumference of affected limb), of which the parameters were continuous, was performed with the fixed effect model and Inverse Variance method. Random effect model was used if subgroup and sensitivity analyses cannot settle heterogeneity issue. The standard mean difference (SMD, which standardizes the results of the studies to a uniform scale so that they can be combined), 95% CI and *p* value were calculated. *p*<0.05 was considered statistically significant.

## Results

### Characteristics of included studies and quality assessment

**Fig 1:**
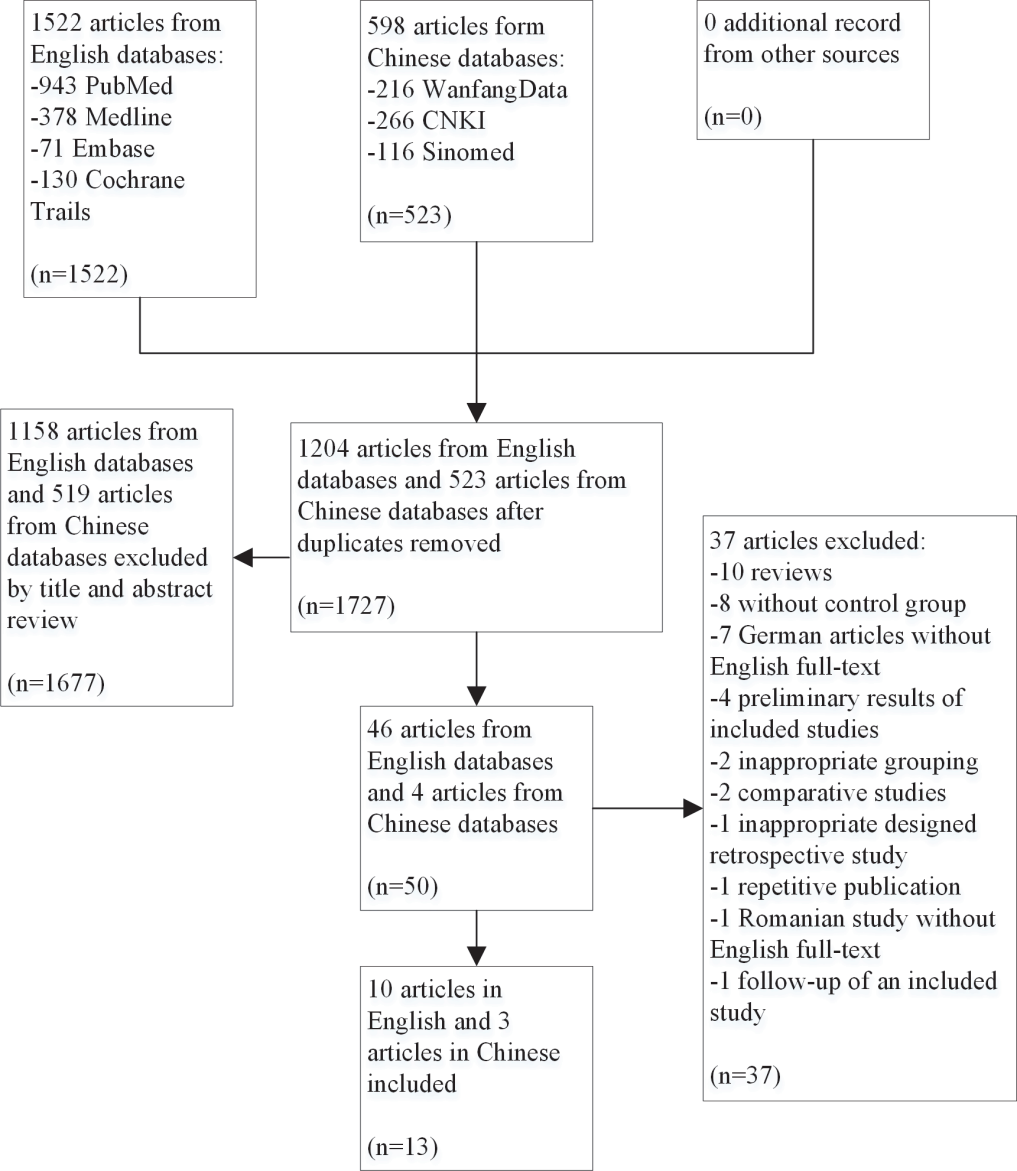
Flow diagram of study selection.

The details of literature search strategy are provided in the supporting information (S1 Table). 1204 articles from English databases and 523 articles from Chinese databases were retrieved. After screening the titles and abstracts, 50 articles remained. When carefully examining the full texts, we excluded another 37 articles for reasons listed in the supporting information (S2 Table). Finally we included 13 studies for the meta-analysis, with 10 in English and 3 in Chinese, and a total of 3269 patients. (Fig. 1)

All the studies were RCTs or cohort studies with reasonable design. The primary endpoints of all the recruited studies consisted of one or multiple of the followings: symptomatic PE, PE detectable by CT or scintigraphy, progression of DVT or DVT related deaths. 7 articles provided proper results of the secondary endpoints suitable for the meta-analysis pertaining to the extent of pain or edema of affected limb. The main characteristics of included studies are shown in Table 1. Patients in bed rest group were kept in bed for at least 3 days, and early ambulation group started exercising within 3 days after diagnosis of DVT. None of the studies found an increased incidence of PE or progression of DVT in the ambulation group.

**Table 1:** Main characteristics of 13 studies included in the meta-analysis.

Study/author year	Country of study	Study design	Patients Male no./Female no. / Mean Age ± SD (years)	Intervention		Primary endpoints	Outcome(end events/total patients)		*P*
				Anticoagulation regimen	Bed rest duration /ambulation date		bed rest	ambulation	
Schellong 1999 [8]	German	RCT	No details	LMWH+Warfarin	8 days /day 0	PE assessed by serial ventilation /perfusion SPECT in 8~10 day	10/59	14/63	0.25
Ashwanden 2001 [9]	Switzerland	RCT	72/57/65±17	LMWH+VitK antagonist	4 days /day 0	PE detected by scintigraphy in 3 months	6/60	10/69	0.44
Blattler 2003 [4]	Austria	RCT	No details	LMWH+VitK antagonist	9 days /day 0	Progression of DVT	4/10	6/27	<0.01^a^
Trujillo-Santos 2005 [5]	Spain	Prospective study	1118/920/No details^b^	LMWH	≥3 days /No details	Symptomatic PE in 15 days	7/1050	4/988	NS
Junger 2006 [3]	German	RCT	57/45/60.4±14.5	Dalteparin + ph enprocoumon	≥5 days /day 0	Combined^c^	14/50	7/53	0.088
Romera 2006 [10]	Spain	RCT	78/68/60.7	LMWH+Warfarin	5 days /day 0	Symptomatic PE during first 10 days	2/67	2/79	0.33
Isma 2007 [11]	Sweden	RCT	39/33/54±14	LMWH+Warfarin	No details /immediately	recanalization of occluded vein within 6 months	-	-	NS
Romera 2008 [12]	Spain	RCT	118/101/64.2	LMWH+Warfarin	5 days /day 0	Symptomatic PE during first 10 days	2/105	3/114	0.54
Manganaro 2008 [13]	Italy	Retrospective case-control study	118/134/65±17	LMWH+oral anticoagulation^d^	7±2 days or permanently /day 0	Combined^c^ at 30-day follow-up visit	43/80	18/172	<0.001^a^
Rahman 2009 [14]^e^	Turkey	RCT	17/7/52.08	Unfractionated heparin/LMWH+Warfarin	7 days /day 0	Improvement of venous outflow at day 7	-	-	NS
Huang 2010 [15]	China	RCT	21/19/61	LMWH+Warfarin	7~10 days /d 1~2	Symptomatic PE during 3 months	0/20	0/20	NS
Feng 2011 [16]	China	nRCT	11/21/60.5	LMWH+Warfarin	7 days /day 0	PE(symptomatic or detectable by CT) in 7 days	1/17	1/15	>0.05
Liu 2013 [17]	China	RCT	38/22/57.0	Routine anticoagulation^d^	7~14 days /day 1~2	symptomatic PE during 3 months	0/30	0/30	NS

^a^ Favors ambulation.

^b^ Partial data eligible for the meta-analysis was extracted. For this reason the details about mean age of these patients were not available.

^c^ Combined endpoints: progression of DVT documented by duplex sonography or phlebography, new PE detected by scintigraphy or CT.

^d^ No detail was mentioned in the article.

^e^ Considering the grouping design, we only included partial data of the study (Group A and Group C).

NS not significant; RCT randomized controlled study; nRCT non-randomized controlled study; ECG electrocardiogram.

**Fig 2:**
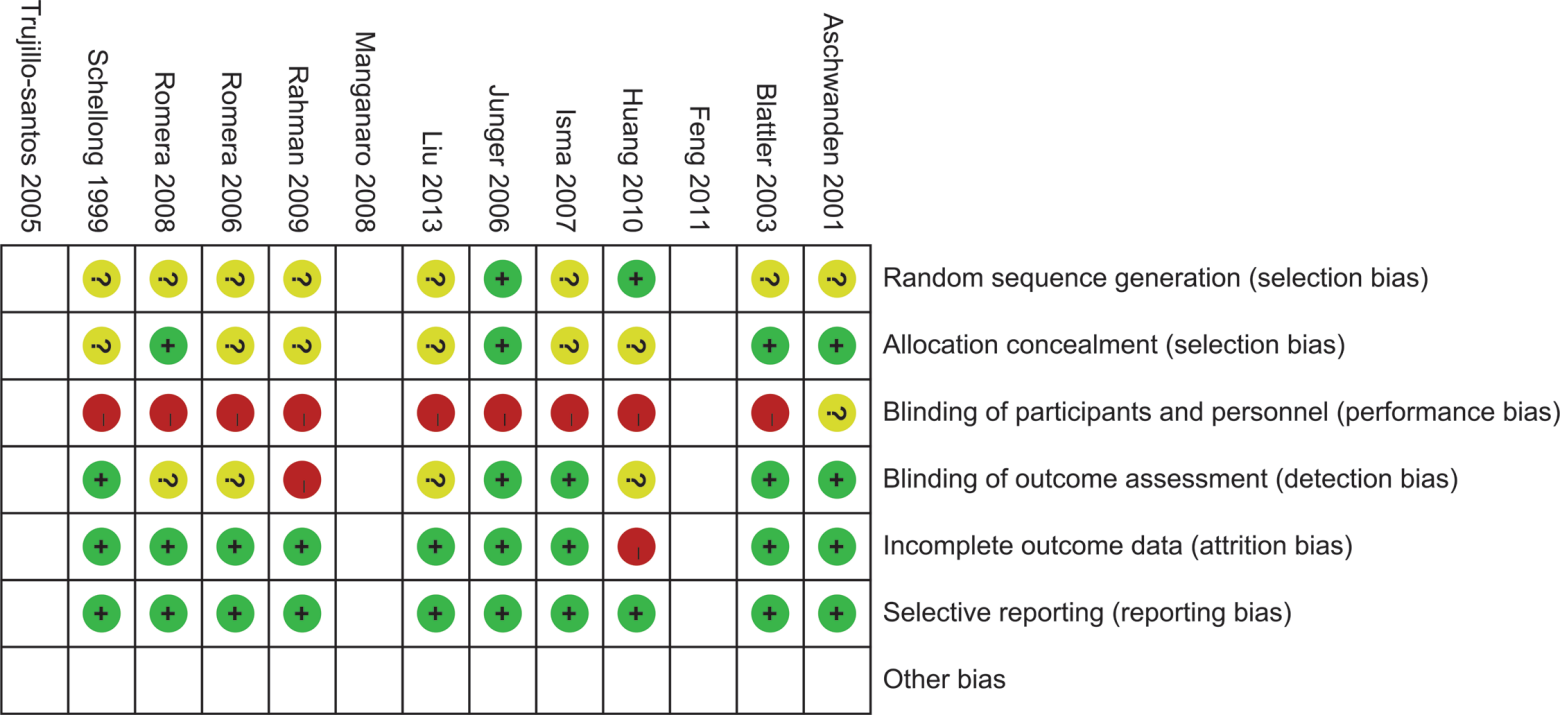
Summary of risk of bias for RCTs (RevMan 5.3). Red: High risk; Yellow: Unclear risk; Green: Low risk * Because the studies compared bed rest versus ambulation, it is too easy for the patients to find out which group they are in. So it is not likely to fulfill the requirement of blinding of participants. ** Details about the reasons for this assessment are listed in the supporting information S3 Table.

Quality assessment of included studies was conducted with the Risk of bias table in RevMan 5.3 for RCTs (Fig. 2 and S3 Table) and Newcastle-Ottawa Scale for n-RCTs (Table 2).

**Table 2:** Quality assessment of cohort studies based on the Newcastle-Ottawa Scale (range, 1–9 stars).

Study	Selection				Comparability		Outcome			Total
	Representativeness of exposed cohort	Selection of non-exposed cohort	Ascertainment of exposure	Demonstration that outcome was not at the start	Control for main factor	Controls for additional factor	Assessment	Follow-up was long enough	Adequacy of follow-up	
Trujillo-Santos 2005 [5]	1	1	0	1	1	0	1	1	0	6
Manganaro 2008 [13]	1	1	0	1	0	0	1	0	0	5
Feng 2011 [16]	1	1	0	1	1	0	0	0	1	5

### Publication bias assessment

**Fig 3:**
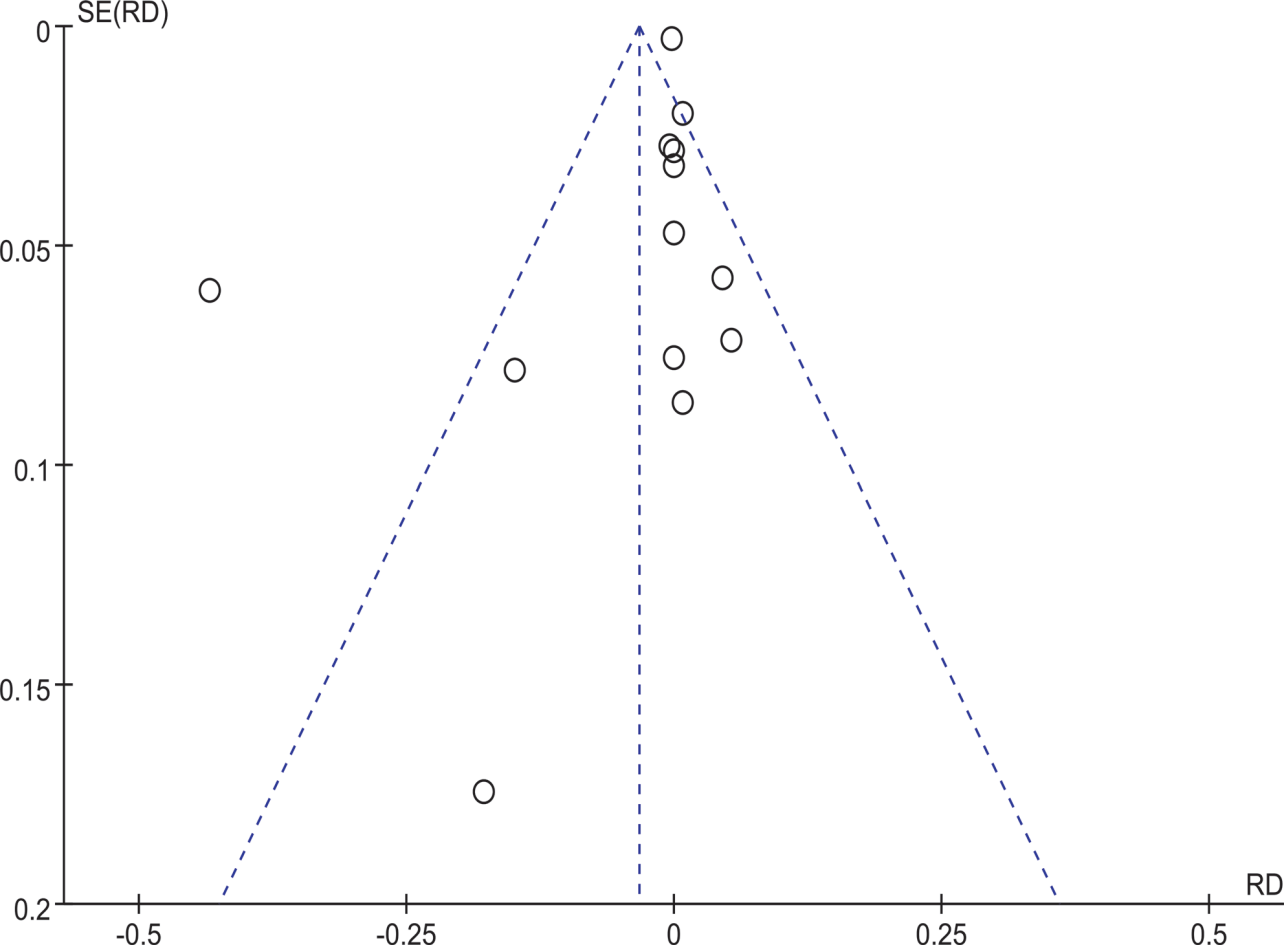
Funnel plot of studies included.

**Fig 4:**
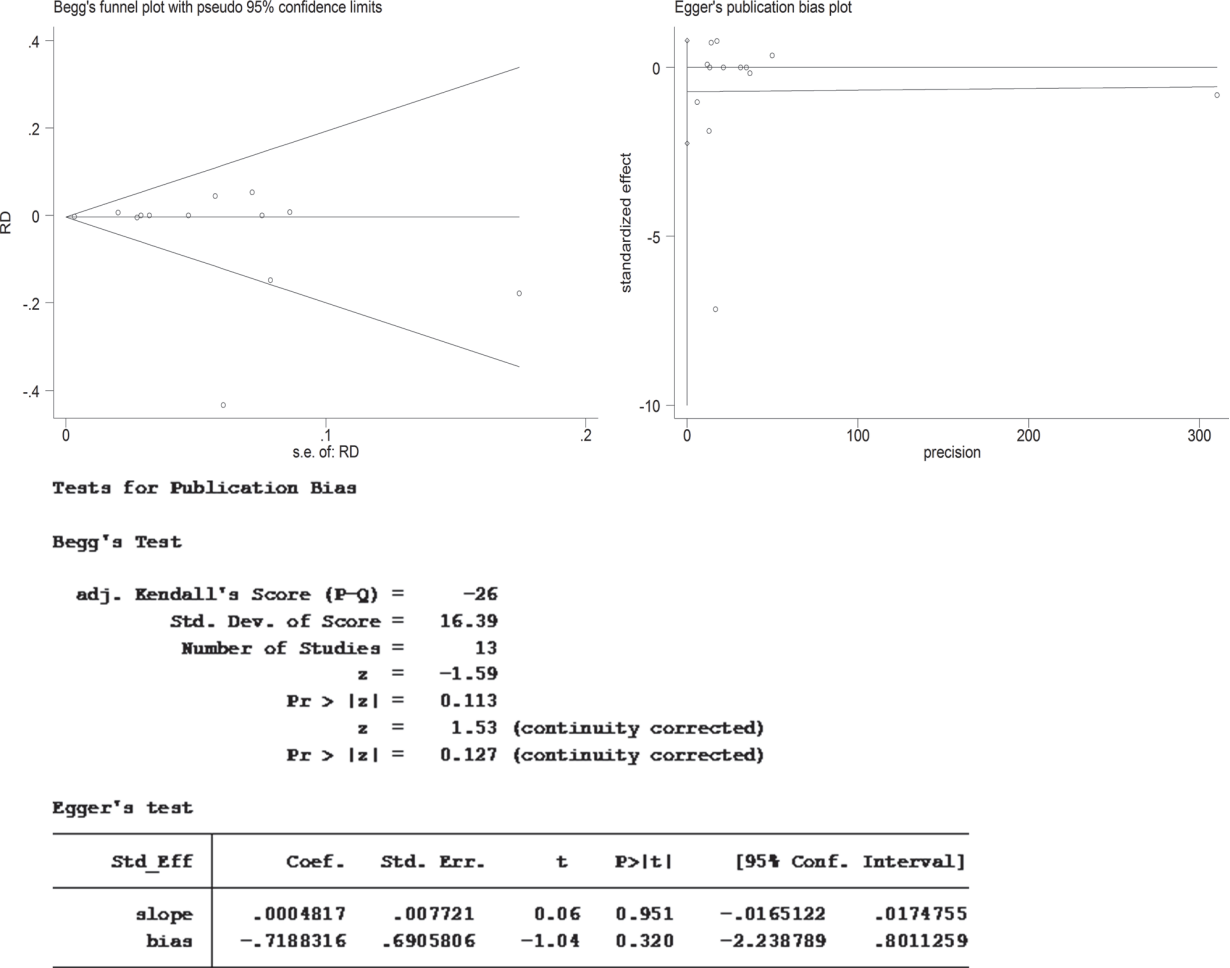
Begg’s and Egger’s tests for publication bias of included studies.

We assessed the publication bias by visual inspection of funnel plot (with RevMan 5.3) as well as Begg’s and Egger’s tests (with Stata 12.1). With visual inspection, there seem to be asymmetry in the funnel plot, indicating the possibility of publication bias (Fig. 3). To further address this problem, we also performed Begg’s and Egger’s tests, and found no significant publication bias (*p* = 0.127 in Begg’s test and *p* = 0.320 in Egger’s test) (Fig. 4). So it was likely that there were other sources of asymmetry (e.g., poor methodological quality, true heterogeneity, artefact, chance), which led to the false publication bias in the funnel plot, as illustrated in Table 10.4.a of the Cochrane Handbook for Systematic Reviews of Interventions [18].

### Meta-analysis of primary endpoints

**Fig 5:**
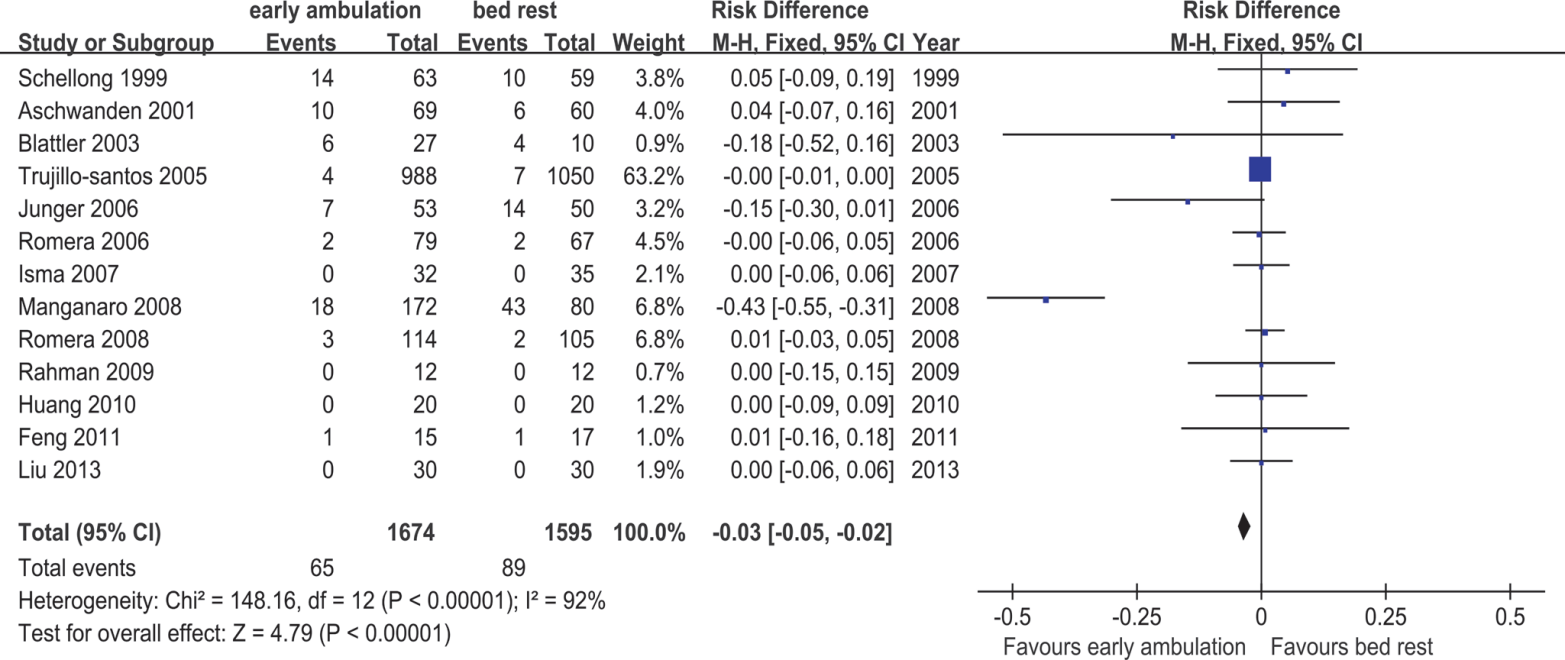
Meta-analysis of the incidence of primary end events among 1674 DVT patients with early ambulation and 1595 DVT patients with bed rest.

Fig. 5 shows the meta-analysis about the incidence of primary endpoints from 13 included studies. As other studies included only DVT patients, with PE patients excluded, we extracted partial data pertaining to DVT patients from Trujillo-Santos’s registry [5]. RD was employed as the effect measure, since no end events happened in 4 of the studies. The heterogeneity test (Chi^2^ = 148.16, *p*<0.00001, I^2^ = 92%) indicated a significant heterogeneity among these studies. Then subgroup and sensitivity analyses were conducted to find the source of heterogeneity (Table 3).

**Table 3:** Summary of subgroup and sensitivity analyses of primary endpoints.

	Number of studies	Heterogeneity			RD (95% CI)	Effect size	*p*_*2*_
		Chi^2^(for FE*) or Tau^2^(for RE**)	I^2^	*p*_*1*_			
Total studies (FE)	13	148.16	92%	<0.00001	−0.003 (−0.005, −0.002)	4.79	<0.00001
Total studies (RE)	13	0.01	-***	-***	−0.03 (−0.05, −0.02)	1.24	0.22
Omitting Manganaro’s study [13] (FE)	12	6.30	0%	0.85	−0.00 (−0.02, 0.01)	0.69	0.49
Omitting Trujillo’s study [5] (FE)	12	94.76	88%	<0.00001	−0.09 (−0.12, −0.05)	4.77	<0.00001
Omitting Trujillo’s study [5] (RE)	12	0.01	-	-	−0.094 (−0.12, 0.03)	1.18	0.24
Subgroup analysis							
RCTs (FE)	10	6.42	0%	0.70	−0.01 (−0.04, 0.03)	0.44	0.66
nRCTs (FE)	3	203.30	99%	<0.00001	−0.04 (−0.06, −0.03)	6.64	<0.00001
nRCTs (RE)	3	0.25	-	-	−0.14 (−0.71, 0.42)	0.49	0.62

*FE fixed effect model

**RE random effect model.

***In a random effect model, Tau^2^ should be employed to indicate the heterogeneity rather than I^2^ and *p*_*1*_ value.

Sensitivity analysis was carried out by leaving out one study at a time. *p*_*1*_ evaluates the heterogeneity among included studies. *p*_*2*_ evaluates the statistical significance level between the two interventions. If *p*_*1*_ is less than 0.05 in a fixed effect model, it means the heterogeneity among included studies is significant and the combined result (*p*_*2*_ value) is not solid and convincing. A random effect model should be employed to draw a more conservative and safer conclucion. According to the statistics in this table, we can draw the conclusion that compared to bed rest, early ambulation is not associated with a higher incidence of primary endpoints.

When there is significant heterogeneity among the studies, the result of a meta-analysis will not be conclusive. Subgroup and sensitivity analyses should be conducted to identify the studies that cause the heterogeneity. Or else, a random effect model should be employed to draw a more conservative conclusion. So according to Table 3, it can be elicited that compared to bed rest, early ambulation was not associated with a higher incidence of new PE, progression of DVT or DVT related death (all studies were analyzed with random effect model, Tau^2^ = 0.01; RD-0.03, 95%CI −0.05~−0.02; Z = 1.24, *p* = 0.22). Trujillo-Santos’s study was not the one which caused significant heterogeneity although it weighted 63.2% when using fixed effect model.

### Meta-analysis of remission of pain and edema

**Fig 6:**
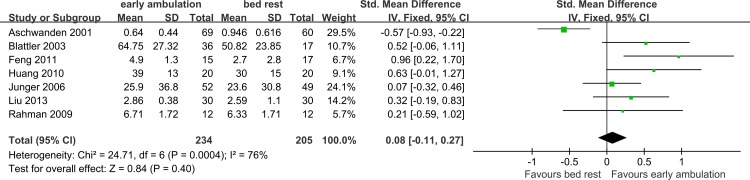
Meta-analysis of VAS change. In this figure, the data in “Mean” and “SD” column is the mean change of VAS rather than initial mean VAS.

Fig. 6 shows the meta-analysis about the remission of limb pain, measured by change of VAS during the treatment period, among the bed rest and early ambulation group. VAS is a continuous and subjective value, therefore the inverse variance method was employed. Due to the different unit system among the studies (e.g., Feng *et al*. used 10 as the highest score [16], while Junger *et al*. used 100 [3]), standard mean difference (SMD) was employed as the effect measure. Using a fixed effect model, heterogeneity test (Chi^2^ = 24.71, df = 6, *p* = 0.0004; I^2^ = 76%) showed significant variance among the studies. Sensitivity analysis indicated that Aschwanden’s study [9] was the main source of heterogeneity (Omitting this study, Chi^2^ = 5.84, df = 5, *p* = 0.32; I^2^ = 14%, fixed effect model).

**Fig 7:**
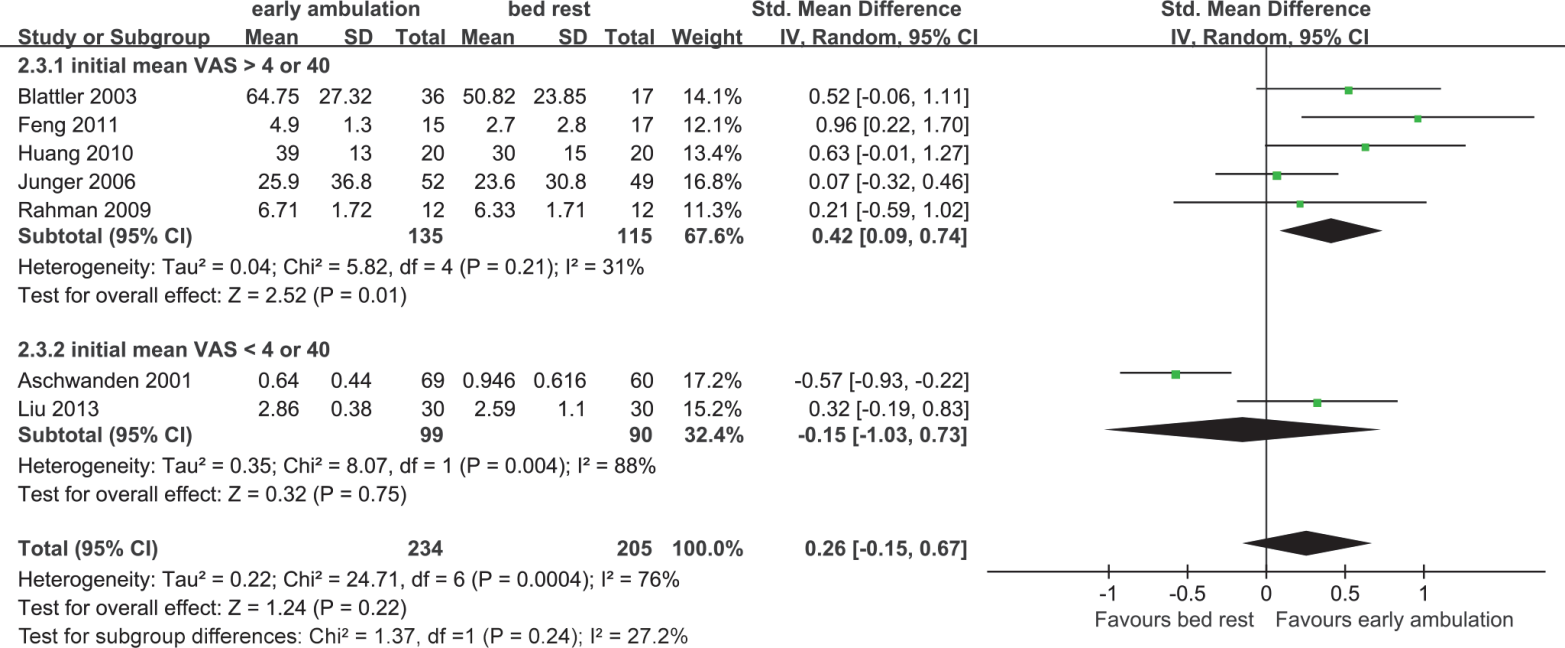
Subgroup analysis of VAS change during the treatment period.

By examining the details of these studies, we found that patients in Aschwanden’s [9] and Liu’s [17] study suffered much milder pain initially (mean VAS 2.23 ± 1.94 for the mobile and 2.82 ± 2.24 for the immobile group in Aschewanden’s study [9], 3.07 ± 0.16 for the mobile and 3.00 ± 0.95 for the immobile group in Liu’s study [17]) than in the other studies (initial mean VAS>4 or 40). Subgroup analysis found that if the patients suffered moderate or severe pain initially, early ambulation was related to a better outcome than bed rest group, in term of reduction of acute pain in the affected limb (SMD 0.42, 95%CI 0.09~0.74; Z = 2.52, *p* = 0.01; random effect model, Tau^2^ = 0.04) (Fig. 7).

**Fig 8:**
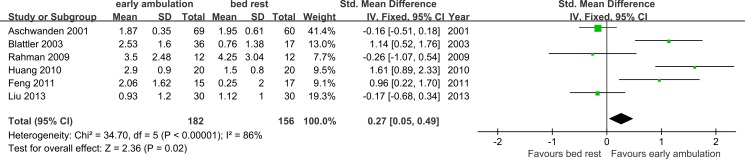
Meta-analysis of change of circumference of affected limb.

Fig. 8 shows the meta-analysis about change of edema with measurement of circumference of affected limb using fixed effect model. Sensitivity analysis (S4 Table) cannot identify any study as the main source of the significant heterogeneity (Chi^2^ = 34.70, df = 5, *p*<0.00001; I^2^ = 86%). So random effect model was employed. Early ambulation was not associated with a better remission of edema of the affected limb (SMD 0.5, 95%CI −0.13∼1.12; Z = 1.55, *p* = 0.12; random effect model, Tau^2^ = 0.51).

## Discussion

The main finding of this meta-analysis was that compared to conventional bed rest treatment, early ambulation was not associated with a higher incidence of PE, progression of DVT or DVT related death in acute DVT patients with effective anticoagulation regimen. It was also associated with a better outcome in term of remission of pain, for patients suffered from moderate or severe pain initially.

There had been 2 English meta-analysis on this topic in 2009, conducted by Aissaoui *et al*. [19] and Anderson *et al*. [20] respectively. Aissaoui *et al*. included 5 studies [3, 5, 8, 9, 21], one of which [21] was interim analysis by Partsch and Blattler published in 2003. Aissaoui *et al*. did not mentioned quality assessment in the article. Anderson *et al*. included 4 studies [3, 4, 8, 9], all of which were included in our meta-analysis. Compared to their analysis, ours included more studies, which made subgroup analysis more feasible. Understandably, our findings were consistent with them, as well as several former reviews [22, 23, 24]. Additionally, we focused on not only PE and prognosis of DVT, but also remission of pain and edema. We also explored whether ambulation could improve recanalization of vessels and reduce Post thrombotic syndrome (PTS), as discussed in the following paragraphs. In 1996, Koopman reported that treatment with LMWH at home was feasible, effective, and safe for patients with proximal vein thrombosis [25]. Included in this meta-analysis, Romera’s study consisted of 2 consecutive RCTs with 365 patients enrolled from January 2002 to December 2007, and proved that it was safe to treat acute DVT at home with early walking, in addition to standard anticoagulation [10, 12]. Hence, it is proper to treat DVT patients as outpatients. Liu *et al*. [17] compared ambulation therapy versus bed rest for acute DVT after stroke among 60 patients, and reported no PE happened in both groups during a 3-month follow-up period. Conversely, Kiser *et al*. [26] and Jiang *et al*. [27] recommended to immobilize the affected limb at least 48–72 hours before an effective anticoagulation was reached. However their studies were retrospective and thus less convincing.

The meta-analysis also found a better remission of acute pain in the affected limb in the early ambulation group, for the patients suffered moderate or severe pain initially. Although it was theoretically reasonable for avoiding one factor of Virchow’s triad, this conclusion should be taken carefully because of the small sample size of each study. In addition, Kahn *et al*. came up with the theory that there was a possibility that exercise may exacerbate pain and edema due to active hyperemia and venous obstruction, which could increase capillary pressure and promote fluid transfusion from capillaries into interstitial space [28]. Isma *et al*. [11] reported that no benefit of early exercise was seen regarding faster remission of pain or swelling. However, there were also other studies that found the opposite. Partsch *et al*. [21] reported that the rate of remission of pain was significantly faster when the patients ambulated with compression. Ratiu *et al*. [29] conducted a RCT with 32 pregnant women diagnosed with proximal deep vein thrombosis and found pregnant women also benefited from leg compression and early mobilization for a faster alleviation of the signs and symptoms of DVT.

In terms of edema in acute DVT patients, we found that early ambulation was not associated with a better remission of edema of the affected limb. Several studies [4, 9, 15, 16, 17, 29] reported a positive effect while others [11, 30] did not. However, it should be noticed that no exacerbation of edema was ever reported with early ambulation.

PTS of the leg arose in one third of patients with first occurred proximal deep vein thrombosis who received standard treatment with anticoagulants [1]. Partsch *et al*. [30] followed up 53 patients with DVT for 2 years, assessing the PTS-score with the Villalta-Prandoni-scale. A significantly better outcome could be found in the mobile group (mean score was 5.1) than in the bed rest group (mean score was 8.2), *p*<0.01. However, this result could be controversial. In Partsch’s study, patients in bed rest group wore no compression stockings while the mobile patients did. It had been reported that the incidence of PTS in patients with first proximal deep vein thrombosis can be reduced by a below-knee graduated elastic compression stocking [1].

Isma *et al*. [11] explored if physiotherapist supervised exercise lasting for 6 months could improve recanalization of thrombotic veins. No benefit of early exercise was seen regarding the degree of recanalization during the 6 months follow-up period in their study.

In addition, ambulation could reduce discomfort of patients, e.g., back pain and constipation [3], as well as costs if treated as outpatients.

By now, we can affirm that early ambulation in acute DVT patients with standard anticoagulation regimen is not associated with a higher incidence of PE, progression of DVT or DVT related deaths. It seems that early ambulation is better for relieving the limb pain, edema as well as PTS condition according to current studies, which is also consistent with the Virchow’s theory.

### Study limitations

Our analysis has several limitations mainly because of the small number and sample size of studies on this topic, as well as the variability among them which led to heterogeneity. Firstly, the Trujillo-Santos’s registry had the largest sample size and counted as much as 63.2% of the total weight. These patients were allocated to bed rest group or ambulation group according to his/her doctor’s advice, which definitely affected the matching of risk factors (e.g., age, malignancy, surgery) [31] between the two groups and could lead to significant bias. Nevertheless, as the authors pointed out, their population-based sample reflected the effects in “real-world” clinical care and enhanced the generalizability of the findings [32]. Secondly, protocols of ambulation were not the same among different studies, e.g., Junger *et al*. [3] instructed patients to move around the ward, while Romera *et al*. [12] treated patients as outpatients and instructed them to establish normal in-house activity and walk 2 to 3 hours a day. Thirdly, the choices about when to begin ambulation were not identical, varying from day 0 to day 3 after the DVT diagnosis. The immobilization duration varied from 3 to 14 days and the follow-up period varied from 7 days to 6 months among the studies. Fourthly, the primary endpoints were symptomatic PE in 4 studies, equipment detectable PE in 6 studies and progression of DVT in another 3 studies. The incidence of symptomatic PE was much lower than asymptomatic PE [23]. Thus studies with symptomatic PE as the endpoints had a lower detectable rate which may lead to a false negative result. Fifthly, nearly all the studies excluded PE patients detectable by CT or scintigraphy at inclusion. While PE occurred in up to 50% of patients with proximal DVT [33]. So the result of this meta-analysis cannot be applied to all acute DVT patients, especially for proximal DVT patients.

## Conclusions

Compared to bed rest, early ambulation of acute DVT patients with standard anticoagulation regimen is not associated with a higher incidence of new PE, progression of DVT, and DVT related deaths. Furthermore, a better outcome can be seen with early ambulation, regarding to remission of acute pain in the affected limb, for those suffered moderate or severe pain initially. Still, more studies are needed to confirm the benefit with respect to reduction of edema, incidence of PTS, as well as extent of recanalization of thrombotic veins.

## Supporting Information

S1 PRISMA Checklist PRISMA 2009 (bed rest vs. ambulation for DVT). (DOC)

S1 Table Literature search strategies and results. (DOCX)

S2 Table Reasons for article exclusion. (DOCX)

S3 Table Quality assessment of included studies. (DOC)

S4 Table Sensitivity analysis of remission of edema. (DOCX)
