# Supplementary material for: Bed Rest versus Early Ambulation with Standard Anticoagulation in The Management of Deep Vein Thrombosis: A Meta-Analysis
Source: PLoS One. 2015 Apr 10;10(4):e0121388. doi: 10.1371/journal.pone.0121388 (PMC4393252; doi:10.1371/journal.pone.0121388)
Supplement: S1 PRISMA Checklist — (DOC) [file pone.0121388.s001.doc]

| **Section/topic** | **#** | **Checklist item** | **Reported on page #** |
| --- | --- | --- | --- |
| **TITLE** | | |  |
| Title | 1 | Bed Rest versus Early Ambulation with Standard Anticoagulation in The Management of Deep Vein Thrombosis: A Meta-analysis | 1,2 |
| **ABSTRACT** | | |  |
| Structured summary | 2 | **Introduction:** Bed rest has been considered as the cornerstone of management of deep vein thrombosis (DVT) for a long time, though it is not evidence-based. While, there is growing evidence favoring early ambulation.  **Methods:** Electronic databases including Embase, Medline, PubMed, Cochrane Library, Sinomed, WanFangData and Chinese National Knowledge Infrastructure, were searched with key words of “deep vein thrombosis”, “pulmonary embolism”, “venous thrombosis”, “bed rest”, “immobilization”, “mobilization” and “ambulation”. We considered randomized controlled trials, prospective or retrospective cohort studies that compared the outcome of acute DVT patients managed with early ambulation versus bed rest, in addition to standard anticoagulation. With the Review Manager version 5.3 software, meta-analysis pertaining to the incidence of new pulmonary embolism (PE), progression of DVT, and DVT related death were conducted, as well as the extent of remission of pain and edema. For the corresponding effective measure, p<0.05 was thought to be statistically significant.  **Results:** 13 studies were included with a total of 3269 patients. Compared to bed rest, early ambulation in the treatment of acute DVT patients with anticoagulation was not associated with a higher incidence of new PE, progression of DVT, or DVT related deaths (RD -0.03, 95% CI -0.05~-0.02; Z=1.24, p=0.22, random effect model). Moreover, if the patients suffered moderate or severe pain initially, the early ambulation group was related to a better outcome, with respect to remission of acute pain in the affected limb (SMD 0.37, 95% CI 0.11~0.62; Z=2.81, p=0.005, fixed effect model). Meta-analysis of alleviation of edema cannot elicit a solid conclusion because of significant heterogeneity among the studies.  **Conclusions:** Compared to bed rest, early ambulation of acute DVT patients with anticoagulation is not associated with a higher incidence of new PE, progression of DVT, and DVT related deaths. Furthermore, for the patients suffered moderate or severe pain initailly, a better outcome can be seen in early ambulation group, regarding to the remission of acute pain in the affected limb. | 2-3 |
| **INTRODUCTION** | | |  |
| Rationale | 3 | Strict bed rest had been considered the cornerstone of treatment of deep venous thrombosis (DVT) for a long period, not only because of the concern of possible dislodging thrombosis, but also the confinement to bed with 24 hours unfractionated heparin infusion. However, this treatment was not evidence-based. More and more doctors began to recognize the risk of blood stasis associated with bed rest. Several randomized controlled trials and prospective registries have investigated the outcomes of DVT patients treated with early ambulation or bed rest and showed no worse outcome associated with ambulation. | 3-4 |
| Objectives | 4 | On the basis of the published studies so far, we perform this systematic meta-analysis to further demonstrate the influence of early ambulation versus bed rest on patients with acute DVT. | 4 |
| **METHODS** | | |  |
| Protocol and registration | 5 | This meta-analysis was not registered. We followed the general protocol of conducting a meta-analysis. Literature search, screening, selection and data extraction were performed independently by different authors. Disagreements were discussed and consulted until a consensus was made. The details can be found in “Materials and Methods” of the manuscript. |  |
| Eligibility criteria | 6 | (1) RCTs, prospective or retrospective cohort studies with good methodological design. (2) All participants were in the acute phase of DVT at recruitment. (3) Interventions were “bed rest” versus “early ambulation”, in addition to standard anticoagulation. (4) End points were new PE (symptomatic or asymptomatic PE confirmed with CT scan or scintigraphy), progression of DVT (assessed by ultrasound or phlebography) or other DVT related parameters (e.g. extent of pain and edema). | 4 |
| Information sources | 7 | Literatures published up to November 2014 were searched in the following database: Embase, Medline, PubMed, Cochrane Library, Sinomed, WanFangData and Chinese National Knowledge Infrastructure (CNKI). The last three were used to search Chinese literatures. | 5 |
| Search | 8 | Key words included: “deep vein thrombosis”, “pulmonary embolism”, “venous thrombosis”, “bed rest”, “immobilization”, “mobilization” and “ambulation”. The synonyms in Chinese were searched in Chinese databases. We made the best of the advanced literature retrieval system in Embase and PubMed to filter clinical trials for our study. In addition, references of relative articles were also examined to make sure all the articles relative to our analysis retrieved. For example,  Search history in Medline   | No. | Query | Results | | --- | --- | --- | | 1 | deep vein thrombosis.mp. or Venous Thrombosis/ | 25201 | | 2 | pulmonary embolism.mp. or Pulmonary Embolism/ | 39398 | | 3 | 1 or 2 | 57555 | | 4 | Bed rest.mp. or bed rest/ | 5949 | | 5 | Immobilization/ or immobilization.mp. | 38092 | | 6 | Mobilization.mp. | 41416 | | 7 | Early ambulation.mp. or early ambulation/ | 2576 | | 8 | 4 or 5 or 6 or 7 | 86125 | | 9 | 3 and 8 | 1073 | | 10 | limit 9 to (humans and (clinical conference or clinical trial or congresses or meta analysis or multicenter study or observational study or randomized controlled trial or ”review” or systematic reviews)) | 378 |   Literature search strategies for different databases can be found in the supplementary content uploaded with the manuscript. | 5 and supporting information Table S1 |
| Study selection | 9 | Title and abstract review was first conducted to rule out articles apparently mismatched to our inclusion criteria. Then the articles would be examined thoroughly to determine whether or not they should be included for the meta-analysis according to the inclusion criteria. Reviews, former meta-analysis and opinions about the disease were also kept for useful information. All the screening work was conducted independently by the two authors (Liu and Tao). Disagreements were discussed and consulted until a consensus was made. The reasons for the exclusion of studies were listed in Table S2. | 5 and supporting information Table S2 |
| Data collection process | 10 | We carefully examined all included articles to extract the useful data and its meanings. We also asked Dr. Hugo Partsch from Department of Dermatology, Wilhelminenspital, Vienna, Austria for his original study data because the result of his article do not provided us exact numbers, which was important parameters in our meta-analysis.  The included studies, designs, and outcomes are listed as TABLE 1 in our manuscript. | 5 |
| Data items | 11 | The incidence of primary end point, change of extent of limb pain measured by visual analogue scales (VAS), and change of extent of edema with measurement of circumference of affected limb under different intervention were extracted from eligible studies. These data can be found in TABLE 1 and FIGURE 5-8 of our manuscript. | 20-23 TABLE 1 and FIG. 5-8 |
| Risk of bias in individual studies | 12 | Quality assessment of included studies was conduct with the Risk of bias table in RevMan version 5.3 for RCTs (Fig 2) and Newcastle-Ottawa Scale for n-RCTs (Table 2). | FIG 2, Table 2. and supporting information Table S3 |
| Summary measures | 13 | Risk Differences rather than Risk Ratio was used to compare the incidence of primary end point, because the incidence is zero in one of the studies.  Standard Mean Difference (SMD) is employed as the effect measure for change of pain and edema due to the different unit system among the studies. | 5-6 |
| Synthesis of results | 14 | The software, Review Manager, make it much easier to synthesize the results. Please see FIGURE 5-8. | 5-6 |

Page 1 of 2

| **Section/topic** | **#** | **Checklist item** | **Reported on page #** |
| --- | --- | --- | --- |
| Risk of bias across studies | 15 | We assessed the publication bias by visual inspection of funnel plot (with RevMan 5.3) as well as Begg’s and Egger’s tests (with the software Stata 12.1 edition). | FIG 3, FIG 4 |
| Additional analyses | 16 | Subgroup and Sensitivity analyses were performed with RevMan 5.3. See FIGURES and the corresponding explanation in the “Results” part of our manuscript. | 6 |
| **RESULTS** | | |  |
| Study selection | 17 | Please see FIGURE. 1, the flow diagram. | FIG. 1 |
| Study characteristics | 18 | Please see TABLE 1. | 20-23 |
| Risk of bias within studies | 19 | Quality assessment of included studies was conduct with the Risk of bias table in RevMan version 5.3 for RCTs (Fig 2) and Newcastle-Ottawa Scale for n-RCTs (Table 2). | FIG 2, Table 2. and supporting information Table S3 |
| Results of individual studies | 20 | TABLE 1 | 20-23 |
| Synthesis of results | 21 | Compared to bed rest, early ambulation is not associated with a higher incidence of new PE, progression of DVT or DVT related death (all studies analyzed with random effect model, Tau2=0.01, RD -0.03 (-0.05, -0.02); Z=1.24, p=0.22).  If the patients suffered moderate or severe pain initially, early ambulation was related to a better outcome than bed rest group, in term of reduction of acute pain in the affected limb (SMD 0.37, 95% CI 0.11~0.62; Z=2.81, p=0.005, fixed effect model).  Early ambulation was not associated with a better remission of edema of the affected limb (random effect model, Tau2=0.51; SMD 0.5, 95% CI -0.13~1.12; Z=1.15, p=0.12). | 8-10, TABLE 3-4, FIG 6-8 |
| Risk of bias across studies | 22 | FIG 3, FIG 4 | FIG 3, FIG 4 |
| Additional analysis | 23 | Subgroup and Sensitivity analyses were performed with RevMan 5.3. See FIGURES and the corresponding explanation in the “Results” part of our manuscript. | 8-10, TABLE 3-4, FIG 6-8 |
| **DISCUSSION** | | |  |
| Summary of evidence | 24 | Please see the “discussion” part of the manuscript. | 10-13 |
| Limitations | 25 | Please see the “discussion” part of the manuscript. | 13-14 |
| Conclusions | 26 | Compared to bed rest, early ambulation of acute DVT patients with standard anticoagulation regimen is not associated with a higher incidence of new PE, progression of DVT, and DVT related deaths. Furthermore, a better outcome can be seen with early ambulation, regarding to remission of acute pain in the affected limb, for those suffered moderate or severe pain initially. Still, more studies are needed to confirm the benefit with respect to reduction of edema, incidence of PTS, as well as extent of recanalization of thrombotic veins. | 14 |
| **FUNDING** | | |  |
| Funding | 27 | No funding for our meta-analysis. |  |

*From:*  Moher D, Liberati A, Tetzlaff J, Altman DG, The PRISMA Group (2009). Preferred Reporting Items for Systematic Reviews and Meta-Analyses: The PRISMA Statement. PLoS Med 6(6): e1000097. doi:10.1371/journal.pmed1000097

For more information, visit: **www.prisma-statement.org**.

Page 2 of 2
