# Supplementary material for: Bed Rest versus Early Ambulation with Standard Anticoagulation in The Management of Deep Vein Thrombosis: A Meta-Analysis
Source: PLoS One. 2015 Apr 10;10(4):e0121388. doi: 10.1371/journal.pone.0121388 (PMC4393252; doi:10.1371/journal.pone.0121388)
Supplement: S1 Table — (DOCX) [file pone.0121388.s002.docx]

**Table S3. Literature search strategies:**

Search history in Medline

| No. | Query | Results |
| --- | --- | --- |
| 1 | deep vein thrombosis.mp. or Venous Thrombosis/ | 25201 |
| 2 | pulmonary embolism.mp. or Pulmonary Embolism/ | 39398 |
| 3 | 1 or 2 | 57555 |
| 4 | Bed rest.mp. or bed rest/ | 5949 |
| 5 | Immobilization/ or immobilization.mp. | 38092 |
| 6 | Mobilization.mp. | 41416 |
| 7 | Early ambulation.mp. or early ambulation/ | 2576 |
| 8 | 4 or 5 or 6 or 7 | 86125 |
| 9 | 3 and 8 | 1073 |
| 10 | limit 9 to (humans and (clinical conference or clinical trial or congresses or meta analysis or multicenter study or observational study or randomized controlled trial or ”review” or systematic reviews)) | 378 |

Search history in EMBASE:

| No. | Query | Results |
| --- | --- | --- |
| #1 | 'pulmonary embolism'/mj | 27879 |
| #2 | 'deep vein thrombosis'/mj | 12649 |
| #3 | 'vein thrombosis '/mj | 12931 |
| #4 | #1 OR #2 OR #3 | 49349 |
| #5 | 'bed rest'/mj | 1586 |
| #6 | 'immobilization'/mj | 7070 |
| #7 | 'mobilization'/mj | 2692 |
| #8 | 'early ambulation'/mj | 2692 |
| #9 | #5 OR #6 OR #7 OR #8 | 11210 |
| #10 | #4 AND #9 | 105 |
| #11 | #4 AND #9 AND [humans]/lim AND [embase]/lim | 71 |

Search history in PubMed:

| No. | Query | Results |
| --- | --- | --- |
| 1 | Search ((deep vein thrombosis[MeSH Terms]) OR pulmonary embolism[MeSH Terms]) OR venous thrombosis[MeSH Terms] | 69217 |
| 2 | Search (((bed rest[MeSH Terms]) OR early ambulation[MeSH Terms]) OR mobilization[MeSH Terms]) OR immobilization[MeSH Terms] | 1605868 |
| 3 | Search (((((bed rest[MeSH Terms]) OR early ambulation[MeSH Terms]) OR mobilization[MeSH Terms]) OR immobilization[MeSH Terms])) AND (((deep vein thrombosis[MeSH Terms]) OR pulmonary embolism[MeSH Terms]) OR venous thrombosis[MeSH Terms]) | 1162 |
| 4 | Search (((((bed rest[MeSH Terms]) OR early ambulation[MeSH Terms]) OR mobilization[MeSH Terms]) OR immobilization[MeSH Terms])) AND (((deep vein thrombosis[MeSH Terms]) OR pulmonary embolism[MeSH Terms]) OR venous thrombosis[MeSH Terms]) Filters: Humans | 943 |

Search history in Cochrane Library:

| No. | Query | Results |
| --- | --- | --- |
| #1 | "bed rest":ti,ab,kw or "ambulation":ti,ab,kw or "mobilization":ti,ab,kw or "immobilization":ti,ab,kw (Word variations have been searched) | 5537 |
| #2 | "deep vein thrombosis":ti,ab,kw or "pulmonary embolism":ti,ab,kw or "venous thrombosis":ti,ab,kw (Word variations have been searched) | 4658 |
| #3 | #1 AND #2 | 146 |
| #4 | Limit #3 to Trials | 130 |
