# Supplementary material for: Bed Rest versus Early Ambulation with Standard Anticoagulation in The Management of Deep Vein Thrombosis: A Meta-Analysis
Source: PLoS One. 2015 Apr 10;10(4):e0121388. doi: 10.1371/journal.pone.0121388 (PMC4393252; doi:10.1371/journal.pone.0121388)
Supplement: S2 Table — (DOCX) [file pone.0121388.s003.docx]

**Table S1. Records excluded with reasons.**

| Article/author year | Reasons for exclusion |
| --- | --- |
| 1. Haeger 1969[1] | All patients were mobile. |
| 1. Zemp 1987[2] | This article is in German without English abstract or full-text. |
| 1. Wallois 1989[3] | Review in French. |
| 1. Oburger 1990[4] | This article is in German without English abstract or full-text. |
| 1. Partsch 1991[5] | All patients were mobile. |
| 1. Wuppermann 1991[6] | This article is in German with an English abstract stating that “A simple score for mobilisation of patients with deep venous thrombosis of the leg is proposed. By this score the significantly earlier mobilisation of patients with deep venous thrombosis is possible without risk.” No English full-text. |
| 1. Meyer 1991[7] | This article is in German without English abstract or full-text. |
| 1. Kriessmann 1991[8] | This article is in German without English abstract or full-text. |
| 1. Kechavarz 1995[9] | All patients were mobile. |
| 1. Fiessinger 1996[10] | Review in French. |
| 1. Partsch 1997[11] | All patients were mobile. |
| 1. Schwarz 1998[12] | Preliminary result of Schellong 1999[13]. |
| 1. Partsch 1999[14] | This article is in German with an English abstract stating that “The central problem in connection with "ambulatory treatment" of deep vein thrombosis is the degree of mobility. In future trials it will be essential not only to register carefully anticoagulant therapy but also to define and to measure walking activities and adjuvant compression.” No English full-text. |
| 1. Schellong 1999[15] | Review in German. |
| 1. Stiefelhagen 1999[16] | This article is in German without English abstract or full-text. |
| 1. Schwarz 1999[17] | Repetitive publication of Schellong 1999[13]. |
| 1. Partsch 1999[18] | All patients were mobile. |
| 1. Manganaro 2000[19] | Comparative study in Italian. |
| 1. Partsch 2000[20] | Preliminary result of Blattler 2003[21] |
| 1. Partsch 2001[22] | All patients were mobile. |
| 1. Labas 2001[23] | All patients were mobile. |
| 1. Diehm 2002[24] | This article is in German with an English abstract which implies it is a review. |
| 1. Partsch 2002[25] | Review in English. |
| 1. Fries 2003[26] | Comparative study in German |
| 1. Junger 2003[27] | Preliminary result of Junger 2006[28] |
| 1. Aldrich 2004[29] | Review in English. |
| 1. Partsch 2004[30] | Follow-up of Blattler 2003[21]. The data is not suitable for our analysis. |
| 1. Partsch 2005[31] | All patients were mobile. |
| 1. Partsch 2005[32] | Review in English. |
| 1. Romera 2005[33] | Preliminary result of Romera 2006[34] |
| 1. Feldman 2006[35] | Review in English. |
| 1. Rainfray 2007[36] | Review in French. |
| 1. Ratiu 2009[37] | This is a RCT on pregnant women with an English abstract while a Romanian full-text. We cannot conduct quality assessment. |
| 1. Saab 2010[38] | Evidence based answer to a clinical inquiry.(review) |
| 1. Lozano 2014[39] | This is an analysis of the RIETE registry. It divided the patients into home therapy or in-hospital groups instead of mobilized or bed rest groups. In-hospital is not equal to bed rest. Also the baseline characteristics of the two groups are significantly different. |
| 1. Chu 2013[40] | This study randomized patients into active mobilization and passive mobilization groups, which is not consistent with our eligible criteria. |
| 1. Kiser 1997[41] | This is a retrospective study. The investigator could only use the billing records to determine the time to return to physical therapy rather than the time to mobilization. This could lead to significant bias. |

**References:**

1. Haeger K (1969) Problems of acute deep venous thrombosis. II. Mobilization and Discharge of the patient. Angiology.

2. Zemp E, Widmer LK, Schwob A, Biland L (1987) Lung embolism and anticoagulation in early mobilized thrombosis patients--preliminary results. Vasa Suppl 20: 136-138.

3. Wallois P (1989) [Role of compression and ambulation in the treatment of recent deep venous thromboses]. J Mal Vasc 14 Suppl B: 76-77.

4. Oburger K, Mostbeck A, Partsch M (1990) Mobility of patients with pelvic vein thrombosis. Vasa Suppl 30: 161-163.

5. Partsch H, Oburger K, Mostbeck A (1991) Frequency of pulmonary embolism in pelvic vein thrombosis under coagulation, compression bandages, and walking. Phlebologie 20: 205-209.

6. Wuppermann T, von Nettelbladt E, Schreibweiss M, Richter H, Bork-Wolwer L (1991) Mobilization following deep venous thrombosis of the leg. Vasa Suppl 33: 229-230.

7. Meyer P, Nobbe F (1991) When can a patient with multi-level thrombosis following administration of anticoagulant therapy be mobilized again. Vasa Suppl 33: 231.

8. Kriessmann A (1991) Bed rest in deep venous thrombosis of the legs? Dtsch Med Wochenschr 116: 596.

9. Kechavarz B, Kohn H, Mostbeck A, Partsch H (1995) Prevalence and incidence of pulmonary embolism in deep vein thrombosis treated by compression, ambulation and low-molecular weight heparin. Phlebology'95.

10. Fiessinger JN (1996) Treatment of venous thromboembolic disease. La Revue du praticien 46: 1235-1239.

11. Partsch H, Kechavarz B, Kohn H, Mostbeck A (1997) The effect of mobilisation of patients during treatment of thromboembolic disorders with low-molecular-weight heparin. Int Angiol 16: 189-192.

12. Schwarz T, Schellong S, Kropp J, Prescher Y, Daniel W (1998) Time of mobilization does not influence the incidence of pulmonary embolism (PE) in patients with deep venous thrombosis (DVT). Preliminary results of a prospective randomized trial. Annals of hematology.

13. SM S, T S, J K, Y P, B B, et al. (1999) Bed rest in deep vein thrombosis and the incidence of scintigraphic pulmonary embolism. Thrombosis and haemostasis.

14. Partsch H (1999) "Ambulatory" therapy of deep venous thrombosis of the leg--definition. Wien Med Wochenschr 149: 28-29.

15. Schellong SM, Schwarz T, Schroder HE (1999) Ambulatory therapy for deep leg vein thrombosis? Dtsch Med Wochenschr 124: 810-815.

16. Stiefelhagen P (1999) Thrombosis ABC, 4: Leg and pelvic vein thrombosis. Compression stockings on the leg and out of bed? MMW Fortschr Med 141: 46.

17. Schwarz T, Kropp J, Prescher Y, Beuthien B, Schellong S (1999) Bed rest in the therapy of deep vein thrombosis and lung emboli incidence. Zeitschrift fur Kardiologie.

18. Partsch H (1999) Thrombophlebitis: bed rest or walking exercise? Wiener medizinische Wochenschrift (1946).

19. Manganaro A, Buda D, Calabro D, Tati' L, Consolo F (2000) Physical treatment of deep venous thrombosis: bed rest or mobilization? Minerva Cardioangiol 48: 53-56.

20. Partsch H, Blattler W (2000) Compression and walking versus bed rest in the treatment of proximal deep venous thrombosis with low molecular weight heparin. Journal of vascular surgery 32: 861-869.

21. Blattler W, Partsch H (2003) Leg compression and ambulation is better than bed rest for the treatment of acute deep venous thrombosis. International angiology : a journal of the International Union of Angiology 22: 393-400.

22. Partsch H (2001) Therapy of deep vein thrombosis with low molecular weight heparin, leg compression and immediate ambulation. Vasa 30: 195-204.

23. Labas P, Ohradka B, Cambal M (2001) Could deep vein thrombosis be safely treated at home? Bratisl Lek Listy 102: 458-461.

24. Diehm C, Stammler F, Lawall H (2002) Deep venous thrombosis of the leg. Will it be acute ambulatory therapy in the future? MMW Fortschr Med 144: 32-34, 36-38.

25. Partsch H (2002) Bed rest versus ambulation in the initial treatment of patients with proximal deep vein thrombosis. Current Opinion in Pulmonary Medicine 8: 389-393.

26. Fries R, Bohm M (2003) Deep venous thrombosis--treatment. pp. 999-1002.

27. Junger M, Sannwald G, Steins A, Storiko H (2003) Prospective randomized study comparing mobilization and immobilization of patients with acute venous thrombosis of the leg. Annals of hematology.

28. Junger M, Diehm C, Storiko H, Hach-Wunderle V, Heidrich H, et al. (2006) Mobilization versus immobilization in the treatment of acute proximal deep venous thrombosis: a prospective, randomized, open, multicentre trial. Current medical research and opinion 22: 593-602.

29. Aldrich D, Hunt DP (2004) When can the patient with deep venous thrombosis begin to ambulate?. Physical therapy 84: 268-273.

30. Partsch H, Kaulich M, Mayer W (2004) Immediate mobilisation in acute vein thrombosis reduces post-thrombotic syndrome. Int Angiol 23: 206-212.

31. Partsch H (2005) Ambulation and compression after deep vein thrombosis: dispelling myths. Seminars in vascular surgery 18: 148-152.

32. Partsch H (2005) Immediate ambulation and leg compression in the treatment of deep vein thrombosis. Dis Mon 51: 135-140.

33. Romera A, Vila R, Perez-Piqueras A, Marti X, Cairols M (2005) Early mobilization in patients with acute deep vein thrombosis: does it increase the incidence of symptomatic pulmonary embolism? Phlebology.

34. Romera A, Cairols-Castellote MA, Perez-Piqueras A, Marti-Mestre FX, Bonell-Pascual A, et al. (2006) Early mobilisation of patients with acute deep vein thrombosis does not increase the risk of a symptomatic pulmonary embolism. Angiologia 58: 127-135.

35. Feldman LS, Brotman DJ (2006) When can patients with acute deep vein thrombosis be allowed to get up and walk? Cleveland Clinic journal of medicine 73: 893-896.

36. Rainfray M, Dehail P, Salles N (2007) Complications of immobility and bed rest. Prevention and management. La Revue du praticien 57: 671-676.

37. Ratiu A, Motoc A, Pascut D, Crisan D, Anca T, et al. (2009) Compression and walking compared with bed rest in the treatment of proximal deep venous thrombosis during pregnancy. Revista medico-chirurgicala a Societatii de Medici si Naturalisti din Iasi.

38. Saab R, Stevermer JJ, Meadows S (2010) Q/Should patients with acute DVT limit activity? Journal of Family Practice 59: 50-52.

39. Lozano F, Trujillo-Santos J, Barron M, Gallego P, Babalis D, et al. (2014) Home versus in-hospital treatment of outpatients with acute deep venous thrombosis of the lower limbs. Journal of vascular surgery 59: 1362-1367.

40. Chu Y, Dou Y, Li Y, Zhou Y (2013) Prognosis of calf deep-vein thrombosis after total knee arthroplasty. Journal of Peking University (Health Sciences) 45: 708-710.

41. Kiser TS, Stefans VA (1997) Pulmonary embolism in rehabilitation patients: relation to time before return to physical therapy after diagnosis of deep vein thrombosis. Arch Phys Med Rehabil 78: 942-945.
