# Supplementary material for: Bed Rest versus Early Ambulation with Standard Anticoagulation in The Management of Deep Vein Thrombosis: A Meta-Analysis
Source: PLoS One. 2015 Apr 10;10(4):e0121388. doi: 10.1371/journal.pone.0121388 (PMC4393252; doi:10.1371/journal.pone.0121388)
Supplement: S3 Table — (DOC) [file pone.0121388.s004.doc]

**Supporting information 1: quality assessment of included studies**


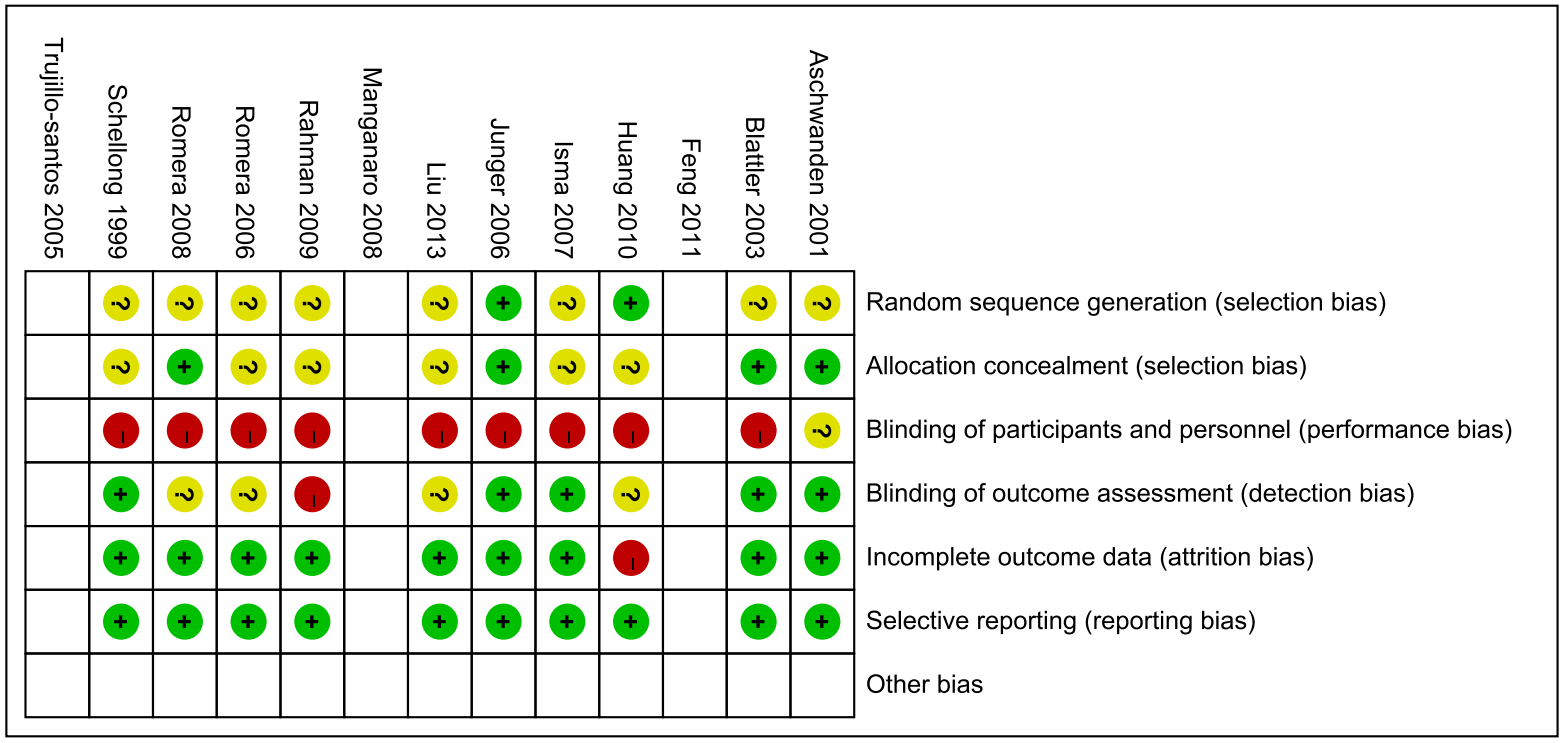


**Fig S1. Assessment of risk of bias of RCTs with the software Review Manager 5.3**

Red: High risk

Yellow: Unclear risk

Green: Low risk

- Because the studies compared bed rest versus ambulation, it is too easy for the patients to find out which group they are in. So it is not easy to fulfil the requirement of allocation concealment or blinding of participants.
- Details about the reasons for this assessment are listed in Table S2.
- Quality assessment of 3 non-RCTs can be found in Table S3 in this document.

**Table S2. Assessment of risk of bias of RCTs with reasons.**

**Schellong 1999**

| **Bias** | **Judgement** | **Support for judgement** |
| --- | --- | --- |
| Random sequence generation (selection bias) | Unclear risk. | Quote: “Patients with acute proximal DVT proven by ultrasonography were randomly assigned to strict bed rest for 8 days (treatment group) or to stay mobilised (control group).”  Comment：Randomization method not mentioned. |
| Allocation concealment (selection bias) | Unclear risk. | Comment: Not mentioned in the article. |
| Blinding of participants and personnel (performance bias) | High risk. | Comment: The allocated intervention is unable to be blinded in this study. |
| Blinding of outcome assessment (detection bias) | Low risk. | Quote: “Lung scans were reviewed by two experienced investigators blinded for treatment groups. Discrepancy of findings was subjected to a consensus process under surveillance of a third investigator who was also blinded for the treatment groups.”  Comment: Probably done. |
| Incomplete outcome data addressed (attrition bias) | Low risk. | 3/62 missing from the bedrest group, 1/64 missing from the mobilized group, because they refused the follow-up lung scan.  Comment: Missing data is relatively small. |
| Selective reporting (reporting bias) | Low risk. | Comment: The study protocol is available and outcomes have been reported in the pre-specified way. |
| Other bias | Unclear risk. | Comment: Insufficient information. |

Aschwanden 2001

| **Bias** | **Judgement** | **Support for judgement** |
| --- | --- | --- |
| Random sequence generation (selection bias) | Unclear risk. | Quote: “The randomization was stratified according to gender and the extension of the thrombosis.”  Comment: Randomization method not mentioned. |
| Allocation concealment (selection bias) | Low risk. | Quote: “used a sealed envelope randomization process to allocate the patients”  Comment: Probably done. |
| Blinding of participants and personnel (performance bias) | High risk. | Comment: The allocated intervention is unable to be blinded in this study. |
| Blinding of outcome assessment (detection bias) | Unclear risk. | Comment: Not mentioned in the article. |
| Incomplete outcome data addressed (attrition bias)  (Short-term outcomes  (Day 4)) | Low risk. | Comment: No missing data. |
| Incomplete outcome data addressed (attrition bias) (Longer-term outcomes  (3 months)) | Low risk. | Quote: “5 patients (3.9%) had died during the 3 months follow-up, three from the ambulating (4.3%) and two from the immobile group(3.3%).”  Comment: Missing data is relatively small. |
| Selective reporting (reporting bias) | Low risk. | Comment: The study protocol is available and outcomes have been reported in the pre-specified way. |
| Other bias | Unclear risk. | Comment: Insufficient information, cannot exclude the possibility of the hospital setting specific selection bias. |

**Blattler, 2003**

| **Bias** | **Judgement** | **Support for judgement** |
| --- | --- | --- |
| Random sequence generation (selection bias) | Unclear risk. | Quote: “randomized trial”  Comment: Randomization method not mentioned. |
| Allocation concealment (selection bias) | Low risk. | Quote: ”sealed envelopes”  Comment: Probably done. |
| Blinding of participants and personnel (performance bias) | High risk. | Comment: The allocated intervention is unable to be blinded in this study. |
| Blinding of outcome assessment (detection bias) | Low risk. | Quote: “on days 0 and 9 the following examinations were done by physicians not aware of the patients’ treatment.”  Comment: Probably done. |
| Incomplete outcome data addressed (attrition bias) | Low risk. | No missing data. |
| Selective reporting (reporting bias) | Low risk. | Comment: The study protocol is available and outcomes have been reported in the pre-specified way. |
| Other bias | Unclear risk. | Comment: Insufficient information. |

Romera, 2006

| **Bias** | **Judgement** | **Support for judgement** |
| --- | --- | --- |
| Random sequence generation (selection bias) | Unclear risk. | Quote: “randomized into two groups.”  Comment: Randomization method not mentioned. |
| Allocation concealment (selection bias) | Unclear risk. | Comment: Not mentioned in the article. |
| Blinding of participants and personnel (performance bias) | High risk. | Comment: The allocated intervention is unable to be blinded in this study. |
| Blinding of outcome assessment (detection bias) | Unclear risk. | Not mentioned in the article. |
| Incomplete outcome data addressed (attrition bias) | Low risk. | No missing data. |
| Selective reporting (reporting bias) | Low risk. | Comment:  The published report includes all expected outcomes. |
| Other bias | Unclear risk. | Comment: Insufficient information. |

Junger, 2006

| **Bias** | **Judgement** | **Support for judgement** |
| --- | --- | --- |
| Random sequence generation (selection bias) | Low risk. | Quote: “using a randomization list”  Comment: Probably done. |
| Allocation concealment (selection bias) | Low risk. | Quote: ”sealed envelope”  Comment: Probably done. |
| Blinding of participants and personnel (performance bias) | High risk. | Comment: The allocated intervention is unable to be blinded in this study. |
| Blinding of outcome assessment (detection bias) | Low risk. | Quote: ”an examiner blinded to the therapy”  Comment: Probably done. |
| Incomplete outcome data addressed (attrition bias) | Low risk. | 1/50 missing from immobile group, for unknown reason.  Comment: Missing data is relatively small. |
| Selective reporting (reporting bias) | Low risk. | Comment: The published report includes all expected outcomes. |
| Other bias | Unclear risk. | Comment: Insufficient information. |

**Isma** 2007

| **Bias** | **Judgement** | **Support for judgement** |
| --- | --- | --- |
| Random sequence generation (selection bias) | Unclear risk. | Quote: “randomly assigned to one of two groups”  Comment: Randomization method not mentioned. |
| Allocation concealment (selection bias) | Unclear risk. | Comment: Not mentioned in the article. |
| Blinding of participants and personnel (performance bias) | High risk. | Comment: The allocated intervention is unable to be blinded in this study. |
| Blinding of outcome assessment (detection bias) | Low risk. | Quote: “The phlebographic scoring was always determined by two investigators blinded regarding treatment.”  Comment：Probably done. |
| Incomplete outcome data addressed (attrition bias) | Low risk. | 1/36 missing from the immobilized group, 4/36 missing from the mobilized group, because of declined participation during study period and refusal of repeat phlebography. |
| Selective reporting (reporting bias) | Low risk. | Comment: The published report includes all expected outcomes. |
| Other bias | Unclear risk. | Comment: Insufficient information. |

**Romera 2008**

| **Bias** | **Judgement** | **Support for judgement** |
| --- | --- | --- |
| Random sequence generation (selection bias) | Unclear risk. | Quote: “patients were randomized into two groups”, ”a computer-generated list”  Comment: Randomization method not mentioned. |
| Allocation concealment (selection bias) | Low risk. | Quote: “closed envelope”  Comment: Probably done. |
| Blinding of participants and personnel (performance bias) | High risk. | Comment: The allocated intervention is unable to be blinded in this study. |
| Blinding of outcome assessment (detection bias) | Unclear risk. | Not mentioned in the article. |
| Incomplete outcome data addressed (attrition bias) | Low risk. | No missing data. |
| Selective reporting (reporting bias) | Low risk. | Comment: The published report includes all expected outcomes. |
| Other bias | Unclear risk. | Comment: Insufficient information. |

Rahman 2009

| **Bias** | **Judgement** | **Support for judgement** |
| --- | --- | --- |
| Random sequence generation (selection bias) | Unclear risk. | Quote: “randomized unblinded controlled trial”  Comment: Randomization method not mentioned. |
| Allocation concealment (selection bias) | Unclear risk. | Comment: Not mentioned in the article. |
| Blinding of participants and personnel (performance bias) | High risk. | Comment: The allocated intervention is unable to be blinded in this study. |
| Blinding of outcome assessment (detection bias) | High risk. | Quote: “randomized unblinded controlled trial”  Comment：Probably not done. |
| Incomplete outcome data addressed (attrition bias) | Low risk. | No missing data. |
| Selective reporting (reporting bias) | Low risk. | Comment: The published report includes all expected outcomes. |
| Other bias | Unclear risk. | Comment: Insufficient information. |

Huang 2010

| **Bias** | **Judgement** | **Support for judgement** |
| --- | --- | --- |
| Random sequence generation (selection bias) | Low risk. | Quote: “patients were randomized by drawing lots”  Comment: Probably done. |
| Allocation concealment (selection bias) | Unclear risk. | Not mentioned in the article. |
| Blinding of participants and personnel (performance bias) | High risk. | Comment: The allocated intervention is unable to be blinded in this study. |
| Blinding of outcome assessment (detection bias) | Unclear risk. | Not mentioned in the article. |
| Incomplete outcome data addressed (attrition bias) | High risk. | 4/20 missing from the immobilized group, 3/20 missing from the mobilized group. |
| Selective reporting (reporting bias) | Low risk. | Comment: The published report includes all expected outcomes. |
| Other bias | Unclear risk. | Comment: Insufficient information. |

**Liu 2013**

| **Bias** | **Judgement** | **Support for judgement** |
| --- | --- | --- |
| Random sequence generation (selection bias) | Unclear risk. | Quote: “randomly divided”  Comment: Randomization method not mentioned. |
| Allocation concealment (selection bias) | Unclear risk. | Not mentioned in the article. |
| Blinding of participants and personnel (performance bias) | High risk. | Comment: The allocated intervention is unable to be blinded in this study. |
| Blinding of outcome assessment (detection bias) | Unclear risk. | Not mentioned in the article. |
| Incomplete outcome data addressed (attrition bias) | Low risk. | No missing data. |
| Selective reporting (reporting bias) | Low risk. | Comment: The published report includes all expected outcomes. |
| Other bias | Unclear risk. | Comment: Insufficient information. |

**Table S3**. Judged study quality based on the Newcastle-Ottawa Scale (range, 1-9 stars)

| **study** | **Selection** | | | | | **Comparability** | | **Outcome** | |  | **Total** |
| --- | --- | --- | --- | --- | --- | --- | --- | --- | --- | --- | --- |
|  | Representativeness of exposed cohort | | Selection of non-exposed cohort | Ascertainment of exposure | Demonstration that outcome was not at the start | Control for main factor | Controls for additional factor | Assessment | Follow-up was long enough | Adequacy of follow-up |  |
| **Trujillo-Santos 2005** | 1 | 1 | | 0 | 1 | 1 | 0 | 1 | 1 | 0 | 6 |
| **Manganaro 2008** | 1 | 1 | | 0 | 1 | 0 | 0 | 1 | 0 | 0 | 5 |
| **Feng 2011** | 1 | 1 | | 0 | 1 | 1 | 0 | 0 | 0 | 1 | 5 |
