# Supplementary material for: Bed Rest versus Early Ambulation with Standard Anticoagulation in The Management of Deep Vein Thrombosis: A Meta-Analysis
Source: PLoS One. 2015 Apr 10;10(4):e0121388. doi: 10.1371/journal.pone.0121388 (PMC4393252; doi:10.1371/journal.pone.0121388)
Supplement: S4 Table — (DOCX) [file pone.0121388.s005.docx]

**Table S4. Sensitivity analysis of remission of edema.**

|  | number of studies | Heterogeneity | | | SMD (95% CI) | Effect size | *p_2_* |
| --- | --- | --- | --- | --- | --- | --- | --- |
|  |  | Chi^2^(for FE^*^) or Tau^2^(for RE^**^) | I^2^ | *p_1_* |  |  |  |
| Total studies (FE) | 6 | 34.70 | 86% | <0.00001 | 0.27 (0.05, 0.49) | 2.36 | 0.02 |
| Total studies (RE) | 6 | 0.51 | -^***^ | -^***^ | 0.5 (-0.13, 1.12) | 1.55 | 0.12 |
| Omitting Aschwanden’s study (FE) | 5 | 24.56 | 84% | <0.00001 | 0.57 (0.28, 0.86) | 3.85 | 0.0001 |
| Omitting blatter’s study (FE) | 5 | 26.01 | 85% | <0.00001 | 0.14 (-0.10, 0.38) | 1.14 | 0.26 |
| Omitting Rahman’s study (FE) | 5 | 32.89 | 88% | <0.00001 | 0.31 (0.08, 0.54) | 2.64 | 0.008 |
| Omitting Huang’s study (FE) | 5 | 20.03 | 80% | 0.0005 | 0.13 (-0.11, 0.36) | 1.06 | 0.29 |
| Omitting Feng’s study (FE) | 5 | 30.96 | 87% | <0.00001 | 0.20 (-0.04, 0.43) | 1.66 | 0.10 |
| Omitting Liu’s study (FE) | 5 | 31.14 | 87% | <0.00001 | 0.37 (0.12, 0.62) | 2.95 | 0.003 |

^*^FE fixed effect model; ^**^RE random effect model; ^***^In a random effect model, Tau^2^ should be employed to indicate the heterogeneity rather than I^2^ and *p_1_* value.

Sensitivity analysis was carried out by leaving out one study at a time. *p_1_* evaluates the heterogeneity among included studies while *p_2_* evaluates the statistical significance level between the two interventions. If *p_1_* is less than 0.05 in a fixed effect model, it means the heterogeneity among included studies is significant and the combined result (*p_2_* value) is not solid and convincing. A random effect model should be employed to draw a more conservative and safer conclucion. According to the statistics in this table, we can only draw the conclusion that early ambulation was not associated with a better remission of edema of the affected limb.
